# Thermal Stability of RNA Phage Virus-Like Particles Displaying Foreign Peptides

**DOI:** 10.1186/1477-3155-9-22

**Published:** 2011-05-24

**Authors:** Jerri C Caldeira, David S Peabody

**Affiliations:** 1 Department of Molecular Genetics and Microbiology, University of New Mexico School of Medicine, and Cancer Research and Treatment Center, Albuquerque, New Mexico, 87131, USA

## Abstract

**Background:**

To be useful for genetic display of foreign peptides a viral coat protein must tolerate peptide insertions without major disruption of subunit folding and capsid assembly. The folding of the coat protein of RNA phage MS2 does not normally tolerate insertions in its AB-loop, but an engineered single-chain dimer readily accepts them as long as they are restricted to one of its two halves.

**Results:**

Here we characterize the effects of peptide insertions on the thermal stabilities of MS2 virus-like particles (VLPs) displaying a variety of different peptides in one AB-loop of the coat protein single-chain dimer. These particles typically denature at temperatures around 5-10°C lower than unmodified VLPs. Even so, they are generally stable up to about 50°C. VLPs of the related RNA phage PP7 are cross-linked with intersubunit disulfide bonds and are therefore significantly more stable. An AB-loop insertion also reduces the stability of PP7 VLPs, but they only begin to denature above about 70°C.

**Conclusions:**

VLPs assembled from MS2 single-chain dimer coat proteins with peptide insertions in one of their AB-loops are somewhat less stable than the wild-type particle, but still resist heating up to about 50°C. Because they possess disulfide cross-links, PP7-derived VLPs provide an alternate platform with even higher stability.

## Background

We recently described a method for peptide presentation on virus-like particles (VLPs) of the RNA bacteriophage MS2, which we believe offers several advantages over other display systems for certain applications [1-3]. Peptides are inserted by recombinant DNA methods into a surface loop of coat protein. When expressed from a plasmid in bacteria, the resulting VLPs display the foreign peptides on their surfaces. Each VLP also encapsidates the mRNA encoding its synthesis, thus enabling recovery of affinity-selected sequences from random sequence libraries by reverse transcription and polymerase chain reaction [2-4]. Like the filamentous phage display technique, MS2 VLP display should be useful for the affinity selection of peptides with binding activity for a wide variety of receptor molecules (e.g. antibodies). Unlike filamentous phages, however, MS2 VLPs readily display foreign peptides at such high densities that they are strongly immunogenic. We are exploiting this capability to develop a vaccine discovery technology in which a single particle serves both for epitope identification and immunization [2,4]. The ability to present affinity-selected peptides to the immune system in the same structural context present during their affinity selection may facilitate the isolation of mimotopes able to elicit a desired antibody response [5,6].

Efficient peptide display on the MS2 VLP depends on the tolerance of coat protein folding and stability to insertions in its AB-loop. Unfortunately wild-type coat protein is poorly tolerant of such insertions, the vast majority giving rise to mis-folded, aggregated or degraded proteins [2]. However, taking note of the physical proximity in the dimer of the C-terminus of one polypeptide chain to the N-terminus of its companion chain, we genetically fused the two subunits to form a so-called single-chain dimer [7]. The genetic fusion of subunits in the single-chain dimer suppresses the defects imparted by AB-loop insertions, as long as they are confined to one half of the dimer, allowing the protein to fold correctly and then assemble into the VLP [1,2]. This is due presumably to the increased thermodynamic stability of the single-chain dimer compared to the wild-type protein. We were curious to know whether the peptide insertions alter the stability of the VLP itself.

## Results

**Figure 1:**
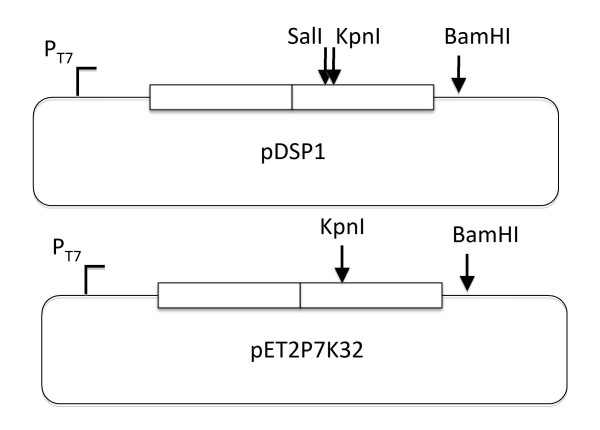
**The plasmids and peptide insertions utilized in this study**. **(A) **pDSP1 expresses the MS2 coat protein single-chain dimer from the T7 promoter. The manipulations that resulted in the various peptide insertions utilized *Sal *I and *Kpn *I sites uniquely present in the downstream half of the dimer. The plasmid pET2P7K32 is a similar construction that expresses the PP7 single-chain dimer.

**Figure 2:**
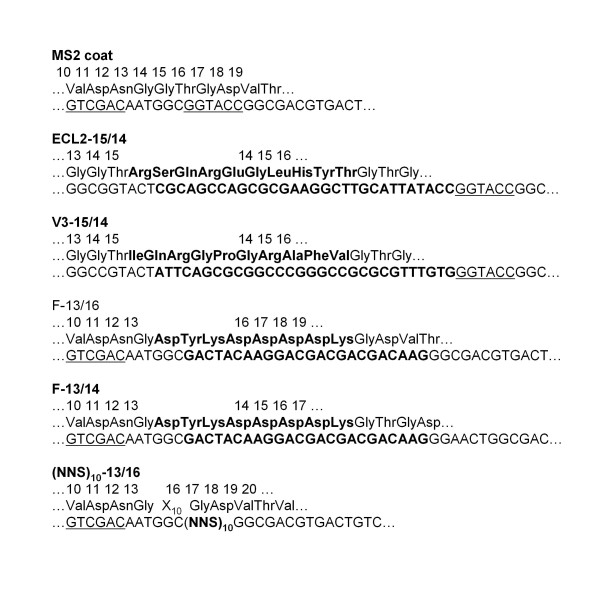
**The amino acid and nucleotide sequences of MS2 coat protein in the vicinity of the various peptide insertions**. Note the positions of *Kpn *I and *Sal *I sites (underlined).

**Figure 3:**
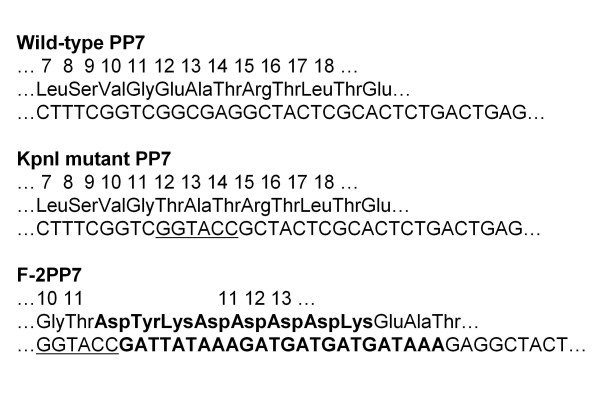
**PP7 sequences, showing the wild-type, the *Kpn *I mutant and the Flag peptide insertion**. Note that the mutations that introduce a *Kpn *I site in the AB-loop and also substitute Glu11 with Thru.

The bacteriophage MS2 coat protein is the major structural protein of the virus and when expressed from a plasmid in *E. coli *it self-assembles into VLPs whose shell structure is virtually identical to that of the MS2 virion. The so-called AB-loop resides on the surface of the VLP and represents a logical site for peptide insertion and display. We previously demonstrated that insertions here generally disrupt coat protein folding/stability, but that genetic fusion of the two dimer subunits suppresses these defects when the insertion is present in the AB-loop of the downstream half of the single-chain dimer [2]. The recombinants described in this paper were created by insertion of several specific foreign peptides, as well as a library of random-sequence peptides, to produce the constructs shown in Figures 1, 2 and 3. VLPs were expressed in *E. coli *and purified by methods detailed previously [8].

**Figure 4:**
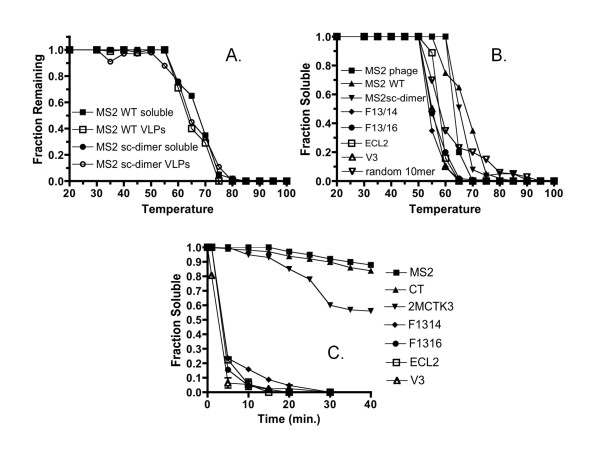
**Denaturation of MS2 VLPs**. **(A) **Coincidence of VLP disassembly, as determined by loss of the VLP band on an agarose gel, and coat protein denaturation, determined by disappearance of soluble protein as a function of temperature. "MS2 WT" denotes the wild-type coat protein, while "MS2 sc-dimer" refers to the MS2 coat protein single-chain dimer. "Soluble" indicates whether the data points were obtained by measuring the amount of soluble protein remaining after treatment and "VLP" identifies data points showing the quantity of intact VLP. **(B) **Denaturation of various recombinant VLPs as a function of temperature. "F13/14" and "F13/16" refer to VLPs displaying the Flag epitope at two different locations in the AB-loop (see the text). "ECL2" and "V3" refer to VLPs displaying the extracellular loop 2 of CCR5 and the V3 loop of the HIV envelope protein, respectively. "Random 10mer" identifies results obtained using a complex library of random-sequence 10mer insertions. **(C) **Denaturation of the indicated MS2 VLPs as a function of time at 55°C.

The denaturation profiles for MS2 wild-type and single-chain dimer VLPs (without an inserted peptide) are plotted in Figure 4a. Note that the values shown in Figure 4 are the averages of two independent measurements. For simplicity, error bars are not shown in the graphs, but the results were highly reproducible, the standard deviations never exceeding a few percent. Of course, it was possible that VLP disassembly might occur at lower temperatures than protein precipitation. To determine the correspondence of the two processes, an aliquot of each of the soluble fractions was subjected to electrophoresis on agarose gel to determine the amount of VLP remaining at each temperature. The coincidence of the curves showing the disappearance of the VLP band from gels, and of coat protein from the soluble fraction suggests that disassembly and denaturation/precipitation are roughly concomitant processes (Figure 4A).

Figure 4B shows the denaturation profiles for wild-type VLPs, single-chain dimer VLPs, and of several VLPs made from single-chain dimers with several specific peptide insertions in their second AB-loops. Under these conditions, wild-type VLPs are half-denatured at about 68°C, while single-chain dimer VLPs (lacking peptide insertions) are slightly less stable, with half-denaturation occurring at around 64°C. The peptide-displaying single-chain dimer VLPs show reduced stability compared to VLPs lacking the peptides. Both versions of the Flag VLP, for example, show half-denaturation at around 53°C. Small differences in the Flag peptide insertion site (Figure 2) do not appreciably alter VLP stability, since F-13/14 and F-13/16 show identical behaviors. The V3 VLP denatures at around 55°C, while the stability of the ECL2 recombinant, at about 57°C, is nearest to that of the parental particle. Thus these AB-loop insertions destabilize the VLP by between 5 and 10°C in this assay. Even so, the particles still possess relatively stable structures that are disrupted only above 50°C.

We determined the time course of VLP denaturation by incubating them for various times at a fixed temperature of 55°C (Figure 4C). Both MS2 phage and wild-type VLPs show slow denaturation at this temperature, losing only about 10% of native protein over the 40 minutes of the experiment. The single-chain dimer VLP is somewhat less stable, with about 60% of the VLP surviving 40. However, the peptide-displaying VLPs denature more rapidly, only 5-20% remaining at the 5-minute time point. This is consistent roughly with the results of the denaturation curves of Figure 4A, since 55°C is close to the melting transitions for each of the recombinant VLPs.

To test the stability effects of peptides generally, we created a complex random-sequence peptide library by insertion of ten NNS triplets between codons for amino acids 13 and 16 in the MS2 AB loop. The denaturation profile of the random 10-mer library (~10^9 ^individual members) is also shown in Figure 4B. The behavior is similar to that shown by the individual recombinants described earlier, with about half the protein precipitating at around 55°C. Here, of course, we are following the average behavior of a highly complex population of VLPs, but it is consistent with the range of behaviors observed for VLPs displaying specific peptides. It should be noted that coat proteins lacking insertions represent about 5-10% of the library population, and probably account for the existence of a second species denaturing at higher temperature. Of course the effects of individual peptide insertions could vary over a wide range and it is even seems possible that some the random sequence peptides have little effect, or may even stabilize the VLP.

### A PP7 recombinant is more stable

We have recently created a system for peptide display on PP7 VLPs similar to the one based on MS2 we described previously [2,3]. As with MS2, we introduced a *Kpn *I site into the PP7 AB-loop-encoding sequence by site-directed mutagenesis. This converted the glutamic acid normally present at position 11 to threonine, but resulted in no apparent alteration of the structure or function of PP7 coat protein as assessed by its translational repressor activity or by its ability to assemble into a VLP (not shown). We constructed a peptide-displaying version of PP7 by inserting the Flag sequence as illustrated in Figure 3.

**Figure 5:**
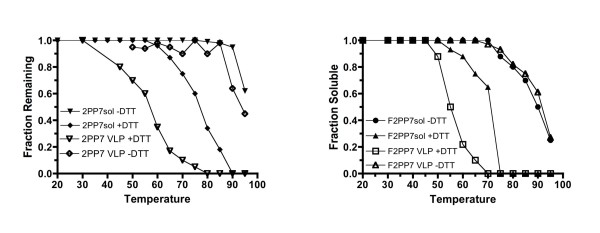
**Comparison of thermal stabilities of PP7 single-chain dimer with and without Flag peptide insertion**. **(A) **Disassembly and aggregation of PP7 single-chain dimer VLPs as a function of temperature in the presence and absence of a reducing agent (DTT). **(B) **Disassembly and aggregation of the Flag-PP7 single-chain dimer VLP with and without DTT.

We showed previously and in Figure 5A, that single-chain dimer PP7 VLPs (without a peptide insertion) are significantly more stable than those of MS2, owing to the presence of inter-dimer disulfide bonds [9]. With the disulfides intact the curves for single-chain VLP disassembly and protein precipitation were roughly coincident, with denaturation and disassembly occurring together above about 85°C. In the presence of DTT, however, we observed a drastic reduction in the temperature at which the VLP disassembled. In fact, when the disufldes were broken it was no more stable than the MS2 single-chain VLP of Figure 4A. However, the PP7 single-chain protein was more resistant to precipitation, so that the curves for VLP disassembly and protein precipitation were not coincident. These results, obtained earlier, are shown for comparison with the results for the Flag-2PP7 recombinant VLPs (Figure 5B). Again the experiment was conducted in both the presence and absence of DTT, and the Flag-2PP7 VLP was assayed for particle disassembly by gel electrophoresis, as well as for protein precipitation. In the absence of DTT, the peptide insertion caused a reduction in VLP stability; the Flag-2PP7 VLP denaturation temperature is roughly 10°C lower than that of its insertion-less counterpart. The curves for protein precipitation and VLP disassembly are similar, indicating that with its disulfide bonds intact, capsid disassembly and protein precipitation occur together. Note also that the Flag-2PP7 recombinant is significantly more stable than the MS2 equivalent, beginning to denature only at temperatures above about 70°C. When DTT is added to the reaction, however, the Flag-2PP7 VLP becomes about as stable as the MS2 recombinant, disassembling at about the same temperature. The protein precipitation curve lags behind, agreeing with our previous result for the single-chain protein lacking insertions. In the case of PP7 genetic fusion of the subunits of the coat protein dimer renders them more resistant to aggregation. Whether this also reflects an increased resistance to thermally induced unfolding, or somehow simply inhibits aggregation of the denatured protein we cannot say. In any case, it is clear that PP7 VLPs are a substantially more stable platform for peptide display than MS2 VLPs, but only when the disulfide bonds are preserved intact.

We note parenthetically that DTT does not detectably alter the stability of MS2's non-disulfide bonded capsid, making it likely that the effect on PP7 is entirely due to reduction of disulfide cross-links (results not shown).

## Discussion

MS2 coat protein folds as a dimer of identical subunits. Its polypeptide chains intertwine in such a manner that the N-terminus of one chain closely approaches the C-terminus of the other. Genetic fusion of the chains yields a single-chain dimer, which we previously showed to be more tolerant of amino acid substitutions and peptide insertions, and more resistant to denaturation than the wild-type dimer [1]. In spite of the increased stability of its dimeric building blocks, however, the single-chain VLP, even without insertions, is actually slightly less stable than the wild-type VLP (Figure 4B). This difference is probably the result of changes in molecular contacts between dimers. In wild-type capsids amino acids near the N- and C-termini participate in capsid-stabilizing interactions, and these are partially disrupted by fusion of the termini.

We previously showed that insertion of foreign peptides in the AB-loop of MS2 coat protein normally disrupt folding, so that little or no functional protein is produced [1,2]. Subunit fusion suppresses these defects, restoring both translational repression and VLP assembly activity to the peptide-displaying coat proteins at the usual growth temperature (37°C). The results presented here demonstrate that subunit fusion allows the production of MS2 VLPs stable up to at least 50°C, the peptide insertions typically destabilizing the VLP by 5-10°C. Since the AB-loop is distant from any sites of intersubunit contact in the capsid, the reduced stability of VLPs containing peptide insertions is presumably the result of effects on the stability of the dimer itself. These effects are dramatic in the wild-type dimer, where AB-loop insertions are seldom tolerated, but subunit fusion imparts an increase in thermodynamic stability sufficient to suppress such defects, at least when the insertion is present on only one of the dimer's AB-loops. The AB-loop consists of three small amino acid residues (Gly13-Gly14-Thr15) that make a tight turn connecting the A and B ß-strands in a hairpin. Such turns are known to frequently stabilize protein structure, and sometimes play an active role in nucleating the formation of secondary structure during folding [10]. Enlarging the loop obviously disrupts the turn's structure and replaces it with a large, presumably flexible segment, thus reducing protein stability. Although a number of studies have noted the reduced yields of viruses or VLPs displaying foreign peptides [11-14], we are aware of only one other reporting the effects of loop insertions on virus-like particle stability: Carreira et. al. showed that insertions in surface loops of a Parvovirus VLP destabilized the particle significantly [15].

The covalent cross-linking of the PP7 VLP by disulfide bonds offers an added measure of stability, and although peptide insertions also exert a destabilizing effect here, the stabilities of both the wild-type PP7 and Flag-PP7 particles are significantly higher that those of the corresponding MS2-based VLPs. This opens the possibility of a new peptide display system based on a more stable VLP platform.

## Conclusions

Insertion of foreign peptides into the AB-loop of wild-type MS2 coat protein normally destabilizes the protein to such an extent that it fails even to fold correctly at the usual bacterial growth temperature of 37°C. Subunit fusion increases the stability of coat protein and thereby suppresses these defects, making possible the formation of VLPs. Insertion of foreign peptides into one AB-loop of the coat protein single-chain dimer typically reduces the stability of MS2 VLPs, but by only about 5-10°C. Similar results were obtained for VLPs derived from a single-chain PP7 coat protein, but, because of disulfide cross-links within the capsid, its VLPs are substantially more stable than MS2's. Clearly, however, both VLP types are stable enough to survive at body temperature.

## Methods

### Plasmids and Proteins

Peptide insertions were introduced by various means into the AB-loop of the downstream half of the MS2 coat protein single-chain dimer in the plasmid pDSP1 (see Figure 1). This plasmid expresses the coat protein single-chain dimer from the bacteriophage T7 promoter, T7 transcription terminator, and the polylinker of pET3d and the kanamycin resistance determinant of and origin of replication of pET9. Note that detailed information about pET3d and pET9, as well as the plasmids themselves, are available from a variety of sources. See, for example, http://www.emdchemicals.com/life-science-research/vector-table-novagen-pet-vector-table/c_HdSb.s1O77QAAAEhPqsLdcab;sid=zyxYAfFoBSdZAbyot4ZKVlmoHC_dHjl8CANmJl6p6hy18OF0zPWlOdhv7thBReaY876LAnR3HC_dHiJrSjOQFTZH?back=true. The plasmid called pET2P7K32 was derived from pET3d by insertion of the PP7 coat protein single-chain dimer sequence. The detailed structures of the plasmids shown in Figure 1, including their nucleotide sequences, are available from the authors upon request. The sequences of the inserted peptides and their sites of insertion within the coat protein AB-loop are detailed in Figure 2. VLPs displaying the ECL2 and V3 peptides (derived from an extracellular loop of the macaque chemokine receptor, CCR5, and from the V3 surface loop of the HIV envelope glycoprotein, respectively) were produced by insertion of synthetic duplex oligonucleotides at a *Kpn *I site previously introduced into the AB-loop sequence with two silent mutations in codons 14 and 15 [2]. Insertion at *Kpn *I duplicates Gly14 and Thr15. We call this the 15/14 insertion-mode since amino acids 15 and 14 respectively flank the N- and C-termini of the inserted peptide. Note that the olignucleotides were designed to preserve the *Kpn *I site on only the 3'-side of the insertion. Two other recombinants contained insertions of the Flag peptide between amino acids 13 and 14 (F-13/14), or between residues 13 and 16 with deletion of 14 and 15 (F-13/16). Briefly, we amplified a coat fragment using a primer that attached a *Sal *I site and the Flag sequence to codon 14 or 16, and a second primer that annealed to the plasmid downstream of a *Bam *HI site near the 3'-end of the coat sequence. We took advantage of a *Sal *I site just upstream of the AB-loop to replace the normal *Sal *I - *Bam *HI of the single-chain dimer sequence with the PCR product. The result was the insertion of the peptide either between amino acids 13 and 14 (the 13/14 mode) or between 13 and 16 (the 13/16 mode).

A library of random-sequence peptides was constructed in the single-chain dimer sequence of a plasmid called pDSP62, which was constructed specifically to facilitate the production of complex random sequence peptide libraries. It is similar in design to pDSP1 (shown in Figure 1), but contains an M13 origin of replication, making possible the production of single-stranded pDSP62 DNA after infection with M13 helper phages. This single-stranded DNA can then be utilized for efficient library construction by the site-directed mutagenesis method of Kunkel et al. [16] as implemented by Sidhu et al. [17]. The upstream copy of the coat sequence is a synthetic version designed to contain the maximum number of silent mutations so that a mutagenic primer can be directed to anneal specifically to the downstream copy. The randomized sequence was inserted into the AB-loop between residues 13 and 16 (see Figure 2).

Plasmid pET2P7K32 (Figure 1) produces the single-chain PP7 coat protein dimer and was constructed from vectors described previously [9,18] It differs from its progenitors mainly by the introduction of a new *Kpn *I site within the AB-loop-encoding sequences of the downstream half of the single-chain dimer. Utilization of a *Kpn *I site for peptide insertion follows the model established previously with MS2 coat protein [2,14]. Figure 3 shows the PP7 sequence in the vicinity of the insertion site. Note that the mutation that introduced the *Kpn *I site also caused a substitution of Glu11 with Thr. To insert the Flag sequence we conducted PCR with a primer that attached sequences for a *Kpn *I site and the Flag peptide to codon 11 of the wild-type PP7 coat sequence. The other primer annealed to plasmid sequences downstream of a *Bam *HI site near the 3' end of the coat sequence. Replacement of the *Kpn *I - *Bam *HI fragment of p2P7K32 with this PCR product resulted in insertion of the Flag peptide between Thr11 and Glu11 (see Figure 2). We call this the 11/11 insertion mode.

All proteins were synthesized in strain BL21(DE3) and VLPs were purified by chromatography on Sepharose CL4B as described before [8,19].

Bacteriophage MS2 was produced by infection of E. coli strain A/λ and was purified by equilibrium density centrifugation on CsCl.

### Thermal Stability Measurements

To compare the thermal stabilities of VLPs we first determined a denaturation profile by measuring the fraction of protein remaining soluble after 2 minutes at a given temperature. Twenty-five ul samples of a VLP solution at a concentration of 1 mg/ml in 10 mM Tris-HCl, 100 mM NaCl, 0.1 mM MgCl2, pH 7.2 were added to preheated tubes at a series of specified temperatures. After 2 minutes the tubes were transferred to ice, where they remained for a few minutes until they were subjected to centrifugation at top speed (~18,000 × g) for 5 minutes in a microcentrifuge. The supernatant fraction was then transferred to a fresh tube and the pellet was redissolved in 25 ul of 8 M urea. Bradford assays determined the amount of protein in both the soluble and insoluble fractions [20]. The values shown are the averages of two independent measurements. For simplicity, error bars are not shown in the graphs, but the results were highly reproducible, the standard deviations never exceeding a few percent. For some samples we also subjected a portion of each soluble fraction to agarose gel electrophoresis to determine whether capsid disassembly was concomitant with protein denaturation/precipitation. The gels were stained with Coomassie Brilliant Blue and scanned with a densitometer.

The relative rates of coat protein denaturation at a fixed temperature of 55°C, were determined by a similar method, again using the Bradford assay [20] to determine the amounts of protein in soluble and insoluble fractions at various times after transfer to the elevated temperature.

## Competing interests

The authors declare that they have no competing interests.

## Authors' contributions

Both authors participated in the planning of experiments. JCC performed the stability measurements. DSP performed the recombinant DNA manipulations, purified VLPs, and prepared the manuscript. Both authors read and approved the final manuscript.
